# Authors reply in response to a letter on “Standardized approach for extubation during extracorporeal membrane oxygenation in severe acute respiratory distress syndrome: a prospective observational study”

**DOI:** 10.1186/s13613-023-01215-9

**Published:** 2023-12-02

**Authors:** Roberto Roncon-Albuquerque, Sérgio Gaião, Francisco Vasques-Nóvoa, Carla Basílio, Ana Rita Ferreira, Alberto Touceda-Bravo, Rodrigo Pimentel, Ana Vaz, Sofia Silva, Guiomar Castro, Tiago Veiga, Hélio Martins, Francisco Dias, Catarina Pereira, Gonçalo Marto, Isabel Coimbra, Juan Ignacio Chico-Carballas, Paulo Figueiredo, José Artur Paiva

**Affiliations:** 1 Department of Emergency and Intensive Care Medicine, São João University Hospital Centre, 4200-319 Porto, Portugal; 2 Department of Surgery and Physiology, UnIC@RISE, Faculty of Medicine of the University of Porto, Porto, Portugal; 3 Department of Medicine, Faculty of Medicine, University of Porto, Porto, Portugal; 4 Department of Internal Medicine, São João University Hospital Centre, Porto, Portugal; 5 Critical Care Department, Hospital Álvaro Cunqueiro, Vigo (Pontevedra), Spain; 6 Department of Infectious Diseases, São João University Hospital Centre, Porto, Portugal

Dear Editor,

We appreciate the valuable and insightful comments from Belletti et al. regarding our article “Standardized approach for extubation during extracorporeal membrane oxygenation in severe acute respiratory distress syndrome: a prospective observational study.” [1] and commend the authors for conducting their recent systematic review on the outcomes of awake extracorporeal membrane oxygenation (ECMO) without invasive mechanical ventilation (IMV) in patients with respiratory failure [2]. In fact, awake ECMO without IMV remains experimental for acute respiratory distress syndrome (ARDS), and candidate selection represents a significant challenge given the high mortality rates observed in patients who require intubation after failing this strategy [1, 2].

We agree that active physiotherapy might provide additional advantages when prioritizing weaning from IMV in ECMO patients with severe ARDS. In our study, weaning from neuromuscular blocking agents and sedation were pursued when circulatory shock, significant bleeding, and acute brain injury were absent. Whenever conscious sedation was achieved and the patient was cooperative, active physiotherapy was performed on a daily basis by an experienced multi-professional team under the supervision of a dedicated intensive care medicine specialist from the ECMO Program. This team had a minimum number of 3 elements and consisted of physiotherapists, critical care rehabilitation nurses, critical care nurses, and ECMO specialists. In our experience, this strategy proved feasible, with infrequent and successfully managed complications, aligning with previous reports on the active mobilization of ECMO patients [3, 4]. Of note, active physiotherapy was maintained without relevant constraints after extubation during ECMO (EXT group), which could also have contributed to the favorable clinical outcomes observed using this clinical strategy.

Regarding the baseline parameters on multi-organ failure, patients weaned from ECMO before liberation from IMV (CTRL group) presented higher Simplified Acute Physiology Score (SAPS) II and Sequential Organ Failure Assessment (SOFA) at Intensive Care Unit (ICU) admission, with no significant differences in the ratio between the partial pressure of oxygen in arterial blood and the fraction of inspired oxygen (PF ratio) and in the Murray Score for Acute Lung Injury when compared to patients in EXT group. This indicates that the CTRL group presents more baseline extrapulmonary organ dysfunctions, in agreement with the ELSO guideline for endotracheal extubation in patients with respiratory failure receiving venovenous ECMO, in which readiness for extubation during ECMO depends on the absence of circulatory shock or multi-organ failure [5].

We agree that the evaluation of right ventricular (RV) function is relevant in risk stratification and management of patients with ARDS, namely with the use of echocardiography for the early detection of acute cor pulmonale [6]. Importantly, in patients with refractory severe ARDS, venovenous ECMO has been shown to decrease pulmonary artery pressure, alleviate RV dysfunction, and resolve acute cor pulmonale-induced circulatory failure [7, 8]. In our centers, focused RV function evaluation in patients with ARDS is typically performed in the context of cardiovascular dysfunction. In our study, awake ECMO was only considered in hemodynamically stable patients, so we did not systematically evaluate RV function to ascertain its role in the prediction of the success of extubation during ECMO support. However, given the established role of positive pressure ventilation in the pathophysiology of RV dysfunction in ARDS [9], it is tempting to speculate that extubation during ECMO (EXT group) could have decreased RV afterload with beneficial effects on the RV function.

Finally, we acknowledge the potential role of the early detection of the Macklin effect (identified on chest CT scans) to tailor the respiratory support of patients with ARDS [10]. However, in our study, chest CT scans were not performed routinely at baseline nor before extubation during ECMO, so we are not able to determine the impact of Macklin effect detection on improving patient selection.

Therefore, future studies involving a systematic evaluation of active physiotherapy, organ-specific dysfunction/failure, RV function, and chest imaging should be able to identify additional prognostic indicators and refine the selection and stratification process for patients who could benefit from ECMO without IMV, ensuring both safety and efficacy.

## Data Availability

The datasets used and analyzed during the current study are available from the corresponding author upon reasonable request.

## References

[1] Roncon-Albuquerque R, Gaiao S, Vasques-Novoa F (2023). Standardized approach for extubation during extracorporeal membrane oxygenation in severe acute respiratory distress syndrome: a prospective observational study. Ann Intensive Care.

[2] Belletti A, Sofia R, Cicero P (2023). Extracorporeal membrane oxygenation without invasive ventilation for respiratory failure in adults: a systematic review. Crit Care Med.

[3] Braune S, Bojes P, Mecklenburg A (2020). Feasibility, safety, and resource utilisation of active mobilisation of patients on extracorporeal life support: a prospective observational study. Ann Intensive Care.

[4] Abrams D, Madahar P, Eckhardt CM (2022). Early mobilization during extracorporeal membrane oxygenation for cardiopulmonary failure in adults: factors associated with intensity of treatment. Ann Am Thorac Soc.

[5] Agerstrand C, Abrams D, Bacchetta M, Brodie D. Endotracheal extubation in patients with respiratory failure receiving venovenous ECMO. 2015. https://www.elso.org/Portals/0/Files/ELSO_ExtubationGuidelines_May2015.pdf.

[6] Mekontso Dessap A, Boissier F, Charron C (2016). Acute cor pulmonale during protective ventilation for acute respiratory distress syndrome: prevalence, predictors, and clinical impact. Intensive Care Med.

[7] Reis Miranda D, van Thiel R, Brodie D, Bakker J (2015). Right ventricular unloading after initiation of venovenous extracorporeal membrane oxygenation. Am J Respir Crit Care Med.

[8] Levy D, Desnos C, Lebreton G (2023). Early Reversal of right ventricular dysfunction after venovenous extracorporeal membrane oxygenation in patients with COVID-19 pneumonia. Am J Respir Crit Care Med.

[9] Ganeriwal S, Alves Dos Anjos G, Schleicher M (2023). Right ventricle-specific therapies in acute respiratory distress syndrome: a scoping review. Crit Care.

[10] Belletti A, Pallanch O, Bonizzoni MA (2023). Clinical use of Macklin-like radiological sign (Macklin effect): a systematic review. Respir Med.

